# Contamination and mortality of leaf-cutting ant workers by the quinone inside inhibitor fungicide after social interactions

**DOI:** 10.1038/s41598-023-32796-x

**Published:** 2023-04-15

**Authors:** Tamires Scudillio, Roberto da Silva Camargo, Tarcísio Marcos Macedo Mota Filho, Carlos Alberto Oliveira de Matos, José Cola Zanuncio, Julian Alberto Sabattini, Luiz Carlos Forti

**Affiliations:** 1 Laboratório de Insetos Sociais-Praga, Departamento de Proteção Vegetal, Faculdade de Ciências Agronômicas, Universidade Estadual de São Paulo (UNESP), Botucatu, São Paulo, 18603-970 Brazil; 2 Instituto de Ciências E Engenharia, Universidade Estadual de São Paulo (UNESP), Itapeva, São Paulo, 18409-010 Brazil; 3 Departamento de Entomologia BIOAGRO, Universidade Federal de Viçosa, Viçosa, Minas Gerais 36570-900 Brazil; 4 Department of Ecology, School of Agriculture Science, National University of Entre Rios, National Council for Scientific and Technical Research, Route No. 11 Km 10.5, 3100 Oro Verde, Argentina

**Keywords:** Ecology, Evolution, Zoology, Ecology

## Abstract

Leaf-cutting ants of the genera *Atta* and *Acromyrmex* (Hymenoptera: Formicidae) are the most important pests in forest and agricultural plantations and livestock. Toxic baits are the main method to manage these insects. The objective was to determine whether the behavior of allogrooming, touch, and self-grooming among *Atta sexdens rubropilosa* Forel, 1908 (Hymenoptera: Formicidae) workers disperse the fungicide quinone inside inhibitor and whether this product is toxic to them. This fungicide was applied, topically, in groups of workers and the social interactions between them and their mortality with and without the fungicide were evaluated. The interactions and the quinone inside inhibitor fungicide contamination increased with the number of leaf-cutting ant workers per group. Excessive touches, with subsequent allogrooming, and self-grooming among the ant workers dispersed the quinone inside the inhibitor fungicide causing 100% mortality and indicating its toxicity to this insect. The hypothesis that social interactions contaminated ant colony mates and the toxicity of the fungicide quinone inside inhibitor to workers of the leaf-cutting ant *A. sexdens rubropilosa* was proven.

## Introduction

Leaf-cutting ants of the genera *Atta* Fabricius, 1805 and *Acromyrmex* Mayr, 1865 (Hymenoptera: Formicidae) are the main pests in forest, agricultural and pasture areas in Brazil and other countries in the Neotropical region^1^. Leaf-cutting ants are managed with toxic baits containing the actives fipronil or sulfluramid, with high efficiencies and economic and operational feasibility^2,3^. These baits are formulated with a mixture of active ingredients dissolved in soybean oil and incorporated in citrus pulp as an attractive substrate^3^.

The dispersion of insecticides by leaf-cutting ants in their colonies must be by trophallaxy^4,5^. Trophalaxy is the distribution of food resources, ingested during foraging, among colony members by oral or anal regurgitation and common in social insects such as ants, bees, termites and wasps^6^. This behavior is important to the exchange pheromones, symbionts, and information between individuals in the colony and the transmission of toxic substances and pathogens^7^. Smaller workers of the leaf-cutting ant *Acromyrmex subterraneus subterraneus* Forel 1911 (Hymenoptera: Formicidae) fed nestmates of similar size or larger, probably by trophallaxy^8^. Workers of the subterranean termite *Coptotermes formosanus* Shiraki, 1909 (Battodea: Rhinotermitidae) dispersed the insecticides cypermethrin, chlordane and chlorpyrifos by trophallaxy^9^. About 50% of the workers in a colony of the leaf-cutting ant *Atta sexdens* Forel 1908 (Hymenoptera: Formicidae) directly contaminated themselves during the processing of ant baits to cultivate its symbiotic fungus^10^.

Trophalaxy is a widely discussed and variable behavior among leaf-cutting ant species^8^. The fluid absorption rates by *Camponotus rufipes* Fabricius, 1775 (Formicidae) and *Pachycondyla villosa* Fabricius 1804 (Ponerinae) workers, which feed on aphid nectar and honeydew, are higher than those of the leaf-cutting ant *Atta sexdens rubropilosa* Forel 1908 (Hymenoptera: Formicidae: Myrmicinae) and of the predators of the *Rhytidoponera* complex^11^. The crop of ants, which collect fluids (nectar) during foraging, such as the genus *Camponotus*, is more developed and adapted to this foraging strategy^11^.

Low rates of fluid absorption by leaf-cutting ants affect other foraging strategies. *Atta sexdens* Forel 1908 (Hymenoptera: Formicidae: Myrmicinae) workers ingested liquids during cutting and processing leaves collected to cultivate their symbiotic fungus and rarely visited nectar sources^12^. Furthermore, leaf-cutting ants ingest liquids during gongylid harvesting in the fungus garden, but their crops are not adapted to store large volumes of liquid^13^. The rate of fluid intake by *C. rufipes* workers was 6.7 µl/min, higher than that of *A. sexdens*, at most, 0.6 µl/min^11^. This indicates that trophallaxy may be infrequent or absent in leaf-cutting ants^14^.

Oral trophallaxy may not disperse insecticides in colonies of leaf-cutting ants and these insects should be intoxicated by direct contact during processing and incorporation of toxic baits into the fungus garden. This can also occur through indirect contact with the active ingredient, during social interactions between workers in the colony^15^. These interactions include hygienic behaviors such as allogrooming, self-grooming and touching, besides contact between contaminated workers or not dispersing the insecticide among colony members ^14,16,17^. This social interaction can be the route of contamination with other substances, such as fungicides? An experiment was carried out to test this hypothesis with a fungicide (toxic to insects). The quinone inside inhibitor fungicide was applied topically on *Atta sexdens rubropilosa* Forel, 1908 (Hymenoptera: Formicidae) workers and the social interactions between them, with this fungicide or not, and the mortality of these insect were evaluated.

## Material and methods

### Colonies studied

Sixty colonies of *A. sexdens rubropilosa* with approximately six months old were collected in March 2020 in the municipality of Botucatu, São Paulo state, Brazil, maintained at the Laboratory of Social Insect Pests of UNESP in Botucatu and used in the experiment. Each colony was kept in a container (length: 15 cm, width: 15 cm and height: 15 cm) with a fungus garden and received *Acalypha* spp. (leaves and stems) at a temperature of 24 ± 2 °C, relative humidity of 80% and a photoperiod of 12 h of light. The handling of plant (*Acalypha* spp. leaves and stems) were carried out in accordance with relevant guidelines and regulations.

### Experiment 1

The hypothesis that interactions between leaf-cutting ants disperse insecticides in their colonies was tested. One μl of quinone inside inhibitor fungicide was applied topically at a concentration of 0.1% (mass/mass) in the pronotum of each *A. sexdens rubropilosa* worker. These workers were placed in 250 ml plastic containers with 1.0 cm of plaster at the bottom and a camera above the container with the ants for 24 h, in a completely randomized experimental design with the treatments: group 1:1–1 worker + 1 worker with quinone inside inhibitor fungicide (0.1%); group 2:1–1 worker + 1 worker with quinone inside inhibitor fungicide (1%); group 3:1–1 worker + 1 worker without quinone inside inhibitor fungicide; group 4:1–4 workers + 1 worker with quinone inside inhibitor fungicide (0.1%); group 5:1–4 workers + 1 worker with quinone inside inhibitor fungicide (1%); group 5:1–4 workers + 1 worker without quinone inside inhibitor fungicide; group 9: 1–9 workers + 1 workers with quinone inside inhibitors fungicide (0.1%); group 9:1–9 workers + 1 worker with quinone inside inhibitor fungicide (1%); group 9:1–9 workers + 1 worker without quinone inside inhibitor fungicide; group 19:1–19 workers + 1 worker with quinone inside inhibitor fungicide (0.1%); group 19:1–19 workers + 1 worker with quinone inside inhibitor fungicide (1%); group 19:1–19 workers + 1 worker without quinone inside inhibitor fungicide.

The *A. sexdens rubropilosa* workers were removed from their colonies and separated by size, based on head width, from 1.2 to 2.2 mm. The pronotum of these workers, which would be contaminated, was marked with a small dot of ink made using a white colored pen (Edding®) with excellent adhesion, fast drying and good visibility^18^. After marking, these workers remained for two hours in 350 ml plastic containers with the edges smeared with Fluon (fluoroethylene resin) to prevent them from escaping. Then, topically, 1.0 μl of the fungicide was applied on each worker's pronotum using a Hamilton microsyringe (5.0 μl) and placed in their respective groups. Self-cleaning, mutual-cleaning and touching frequencies were recorded during 24 h of video recording.

### Experiment 2

The mortality per group of contaminated *A. sexdens* workers was evaluated in an experiment similar to the first. A control treatment, consisting of vegetable oil at a concentration of 1.0%, and two others with the fungicide at 0.1% and 1.0% (mass/mass) were evaluated. After marking and contaminating the worker, each group was placed in a transparent plastic container with a diameter of 7.5 cm and 5.5 cm height, with hermetic lids and, at its base, a 1.0 cm of plaster to maintain moisture. The contaminated worker and its group of uncontaminated ants were kept for 24 h without food. After this period, they received approximately 3.0 g of the symbiont fungus and about 20 minimal workers with a head width of approximately 0.8 to 1.0 mm, as they efficiently cultivate the fungus^19^. Ant mortality was evaluated in the 1st, 2nd, 3rd, 5th, 7th, 9th, 11th, 14th, 17th and 21st days after their contamination^20^.

### Data analysis

A regression model, considering the frequency of each behavior with different fungicide concentrations and *A. sexdens rubropilosa* worker proportions, was applied using the Generalized Linear Model (GLM) with negative binomial variance and logarithmic linkage function according to overdispersion data^21^. A residual deviation (36,887.41,989 and 39,656 with 30 degrees of freedom) were non-significant (*P*-value = 0.18, 0.07 and 0.11) indicating a lack of evidence against this model for the frequency of allogrooming, self-cleaning and touching behaviors. Multiple comparison tests, between group means and fungicide concentrations, were performed using the log odds ratio. The p value was adjusted using Tukey's method to compare a family with four and three estimates, respectively^22^.

Multiple comparisons of survival curves were performed using the Log-Rank test^23^ and the False Discovery Rate^24^ to adjust their *P*-value correction.

The MASS, multicomp, emmeans, survival, survminer, ggplot2 and RColorBrewer packages of the R statistical and graphics programming environment version 4.1.2 used the R Core Software^25^.

## Results

### Experiment 1

The frequency of allogrooming behavior was higher in the 01:04, 01:09 and 01:19 groups than in the 01:01 and similar between the concentrations of quinone inside inhibitor fungicide and the control (Table 1).

**Table 1:** Coefficients (C), intercept (Int.) and estimated values (Est.) (mean + standard error-SE), z.value (Zv) and Pr > z (Pv) of the generalized linear model of the touching, self-grooming and allogrooming frequencies between *Atta sexdens rubropilosa* (Hymenoptera: Formicidae) workers contaminated with quinone inside inhibitor fungicide at 0.1% (0.1%) and 1.0% (1.0%) with the proportions of 04, 09 and 19.

C	Touching			Selfgrooming			Allogrooming		
	Est	Zv	Pv	Est	Zr	Pv	Est	Zr	Pv
Int	2.20^0.17^	12.29	< 2^e−16^	3.45^0.13^	26.34	< 2^e−16^	1.09^0.21^	5.11	3.09^e−07^
0.1%	− 0.32^0.14^	− 2.23	0.0257	0.09^0.12^	0.78	0.435	− 0.20^0.14^	− 1.36	0.172
1.0%	− 0.32^0.14^	− 2.21	0.0269	− 0.17^0.12^	− 1.41	0.156	0.06^0.13^	0.49	0.621
04	1.38^0.20^	6.83	8.06^e−12^	0.77^0.14^	5.18	2.21^e−07^	1.06^0.23^	4.56	4.96^e−06^
09	2.51^0.19^	12.88	< 2^e−16^	1.17^0.14^	7.97	1.58^e−15^	1.30^0.22^	5.77	7.65^e−09^
19	3.47^0.19^	17.98	< 2^e−16^	1.55^0.14^	10.62	< 2^e−16^	1.56^0.22^	7.08	1.38^e−12^

Superscripted values are the standard error of the mean for groups.

**Figure 1:**
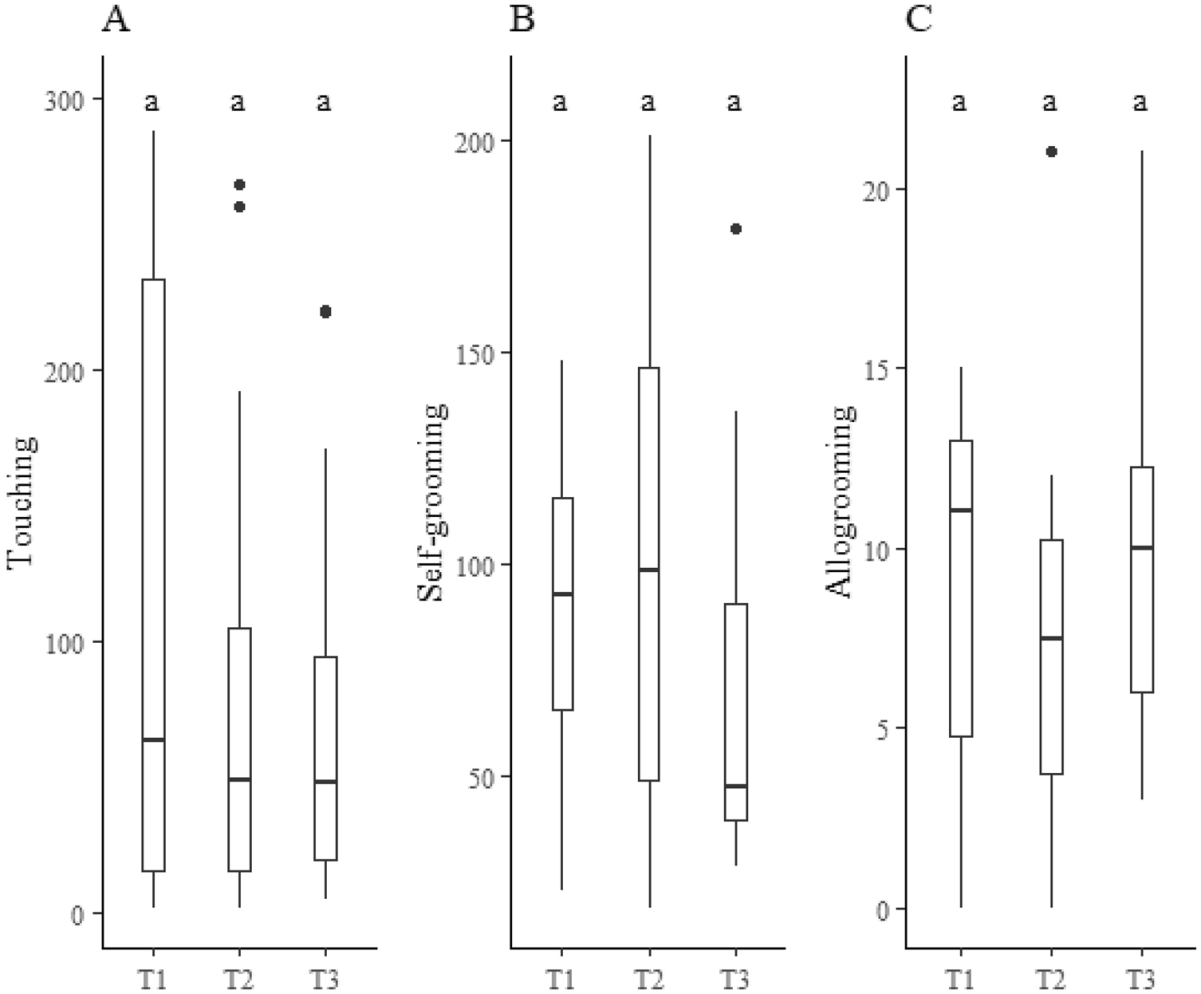
Frequency **boxplot** of behavioral acts of *Atta sexdens rubropilosa* (Hymenoptera: Formicidae) workers in the control (T1), quinone inside inhibitors fungicide 0.1% (T2) and quinone inside inhibitors fungicide 1.0% (T3) treatments. **Mean values** followed by the same letter per behavior do not differ.

The frequency of self-grooming behavior, 01:01, 01:04, 01:09 and 01:19 groups, did not differ between the concentrations of quinone inside inhibitor fungicide and the control (Table 1). The frequency of self-grooming behavior increased, reaching a higher value in the 01:04, 01:09 and 01:19 groups than in the 01:01 (Table 1, Fig. 1), but it did not differ between the concentrations of 0.1%, 1% of quinone inside inhibitors fungicide and the control (Table 1).

**Figure 2:**
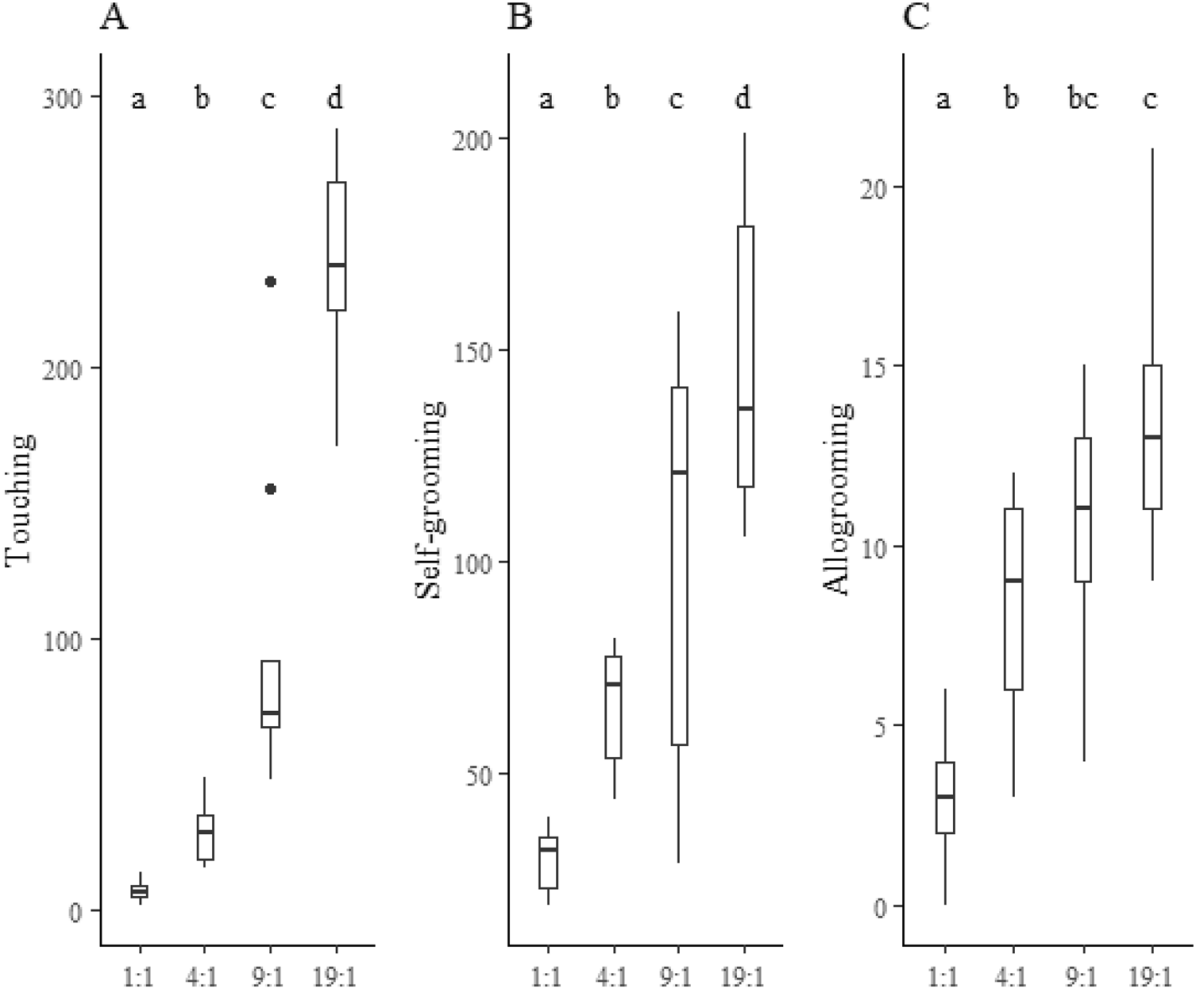
Frequency of behavioral acts of *Atta sexdens rubropilosa* (Hymenoptera: Formicidae) workers in the control (T1), quinone inside inhibitors fungicide 0.1% (T2) and quinone inside inhibitors fungicide 1.0% (T3) treatments. Values ​​followed by the same letter per behavior do not differ.

The frequency of touching behavior was higher in the 01:04, 01:09 and 01:19 groups than in the 01:01 (Table 1, Fig. 1). This frequency was higher with the concentrations of quinone inside inhibitors fungicide at 0.1% and 1% than in the control (Fig. 1). Touching behavior increased with the number of workers of the leaf-cutting ant *A. sexdens rubropilosa* per group (Table 2), but it did not differ between the concentrations of quinone inside inhibitor fungicide 0.1% and 1% and the control (Fig. 2).

**Table 2:** Multiple comparison tests for groups (Gr.) 4:1 (04 vs. 01), 09:01 (09 vs. 01), 09:04 (09. vs. 04), 19:01 (19. vs. 01 ), 19:04 (19. vs. 04) and 19:09 (19. vs. 09) of *Atta sexdens rubropilosa* (Hymenoptera: Formicidae) workers and the values of ratio (Ra.) (Mean + standard error), z.ratio (Zr), *p*.value (pv) on touch, selfgrooming, and allogrooming behaviors.

Gr	Touch			Selfgrooming			Allogrooming		
	Ra	Zr	pv	Ra	Zr	pv	Ra	Zr	Pv
04vs01	3,98^0,80^*	6,83	< .0001	2,17^0,32^	5,18	< .0001	2,89^0,67^	4,56	< .0001
09vs01	12,39^2,41^	12,88	< .0001	3,24^0,47^	7,97	< .0001	3,69^0,83^	5,77	< .0001
09vs04	3,12^0,51^	6,94	< .0001	1,50^0,21^	2,85	0,0223	1,28^0,20^	1,51	0.4259
19vs01	32,38^6,26^	17,98	< .0001	4,74^0,69^	10,62	< .0001	4,78^1,05^	7,08	< .0001
19vs04	8,15^1,31^	12,99	< .0001	2,18^0,30^	5,59	< .0001	1,65^0,25^	3,27	0.0058
19vs09	2,61^0,40^	6,27	< .0001	1,46^0,20^	2,75	0,0299	1,29^0,18^	1,79	0.2742

*Superscripted values are the standard error of the mean (Ra).

### Experiment 2

**Figure 3:**
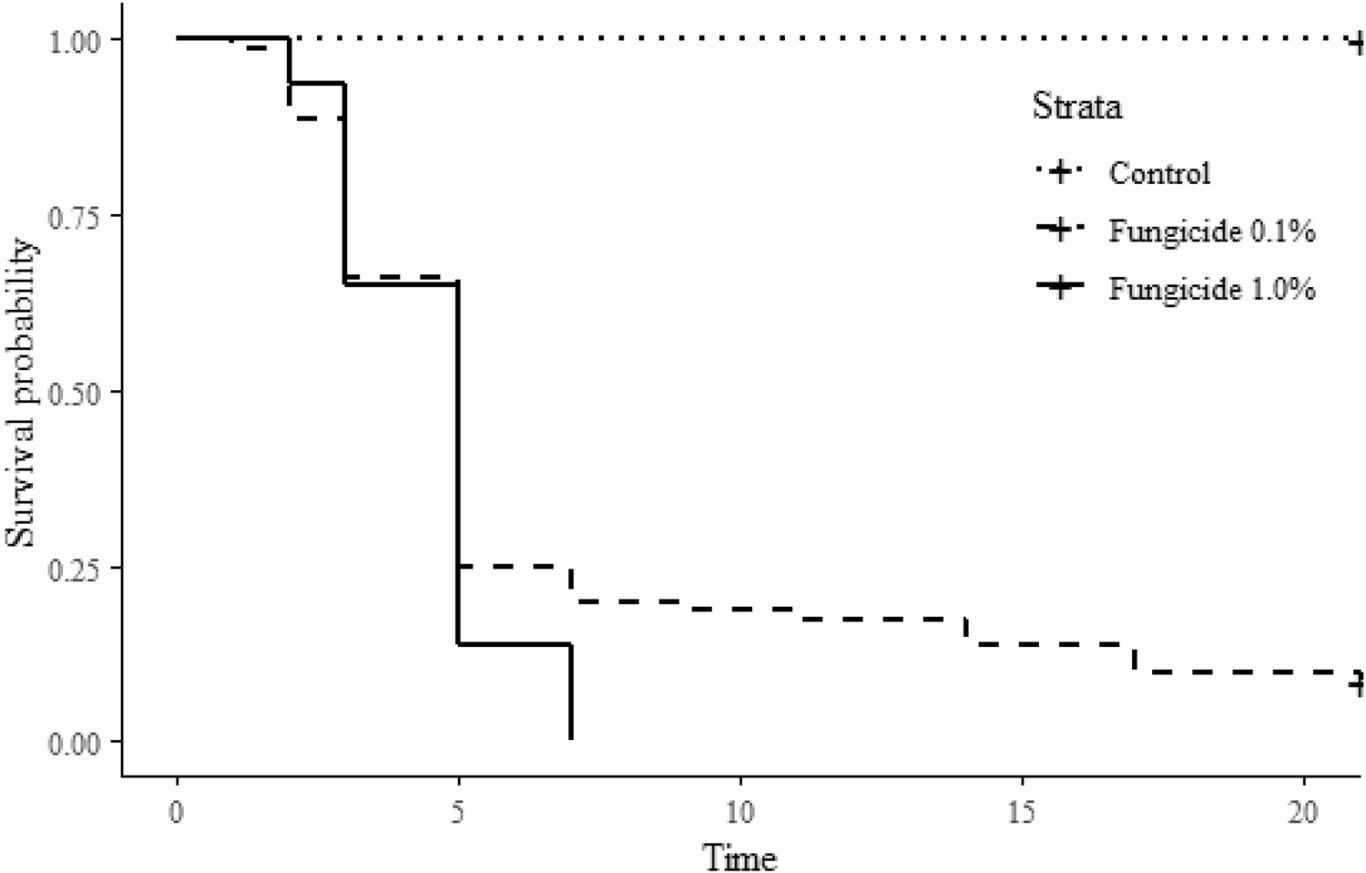
Survival probability curves for *Atta sexdens rubropilosa* (Hymenoptera: Formicidae) workers in groups with the quinone inside inhibitors fungicide 0.1% and 1.0% and in the control.

The survival curves of *A. sexdens rubropilosa* workers, with the concentrations of the fungicide quinone inside inhibitor at 0.1% *p* < 2e-16 and 1.0% *p* < 2e-16) and the control differed from each other (*p* = 0.019) with the death of 100% of the workers with the two concentrations of this fungicide (Fig. 3).

## Discussion

The hypothesis was confirmed that social interactions between A. sexdens rubropilosa workers increase the dispersion of the fungicide quinone inside inhibitor and contaminates others through allogrooming, self-cleaning, and touch behaviors. These results corroborate the higher mortality of ant workers due to the dispersion of fat-soluble substances, including insecticides, in social interactions^15,26^. This mode of action should be similar to that of the sulfluramid insecticide, widely used to manage leaf-cutting ants in Brazil^3^, but that of the latter compound is slower and only by contact^20^.

The higher frequency of allogrooming, self-grooming and touching behaviors among *A. sexdens rubropilosa* workers, after contamination with quinone inside inhibitor fungicide at 0.1% and 1.0%, proves its dispersion by this ant. This dispersion type has been reported for insecticides on different social and semi-social insects such as the Argentine ant, *Linepithema humile* (Mayr) (Hymenoptera: Formicidae)^27–29^ and black carpenter ants, *Camponotus pennsylvanicus* De Geer, 1773 (Hymenoptera, Formicidae) transferring the insecticide fipronil^29^ and the German cockroach *Blattella germanica* (L.) (Blattodea: Blattellidae) the indoxacarb^30^ to nestmates. This occurs mainly when those contaminated at the application place transfer the compound to others in the population^29^ by tarsal or antennal contact in groups or, randomly, by touch between an alive and a dead worker ^27^. The frequency of touching behavior by A. sexdens with workers exposed to the concentrations of quinone inside inhibitor fungicide at 0.1% and 1% was higher than in the control.”

The higher frequency of allogrooming in the 01:04, 01:09 and 01:19 groups than in the 01:01 group. This behavior increases the dispersion of this fungicide due to excessive touching among workers. This is similar to that reported for social interactions among those of *A. sexdens* with allogrooming, self-cleaning, and touching behaviors between contaminated and non-contaminated individuals dispersing the insecticides fipronil and sulfluramid to colony mates^15^. Social interactions disperse insecticidal substances and fungicides among *A. sexdens rubropilosa* workers.

The higher frequency of self-grooming behavior in the groups 01:04, 01:09 and 01:19 is important to remove potentially pathogenic organisms on the surface of the bodies of social insects and from their colony mates^15^. Self-grooming effectively removed parasites such as *Metarhizium* from the ant cuticle^31–33^. The higher frequency of self-grooming behavior in the 01:04, 01:09 and 01:19 groups than in the 01:01 shows an increase in social interactions with the group size and, consequently, the dispersion of insecticidal substances among the colony nests^16,26,34^. Active ingredients, used to manage leaf-cutting ants, act by ingestion and contact and, therefore, hygiene behaviors increase contact with the insecticide and, consequently, ant contamination^3^. On the other hand, the similar frequency of self-cleaning, between the concentrations of quinone inside inhibitors fungicide and the control, demonstrates that the *A. sexdens rubropilosa* workers did not detect this fungicide. This is desirable, as ants, during self-grooming and collective cleaning, ingest particles and substances collected during cleaning, including insecticides, which are absorbed by the post-pharyngeal gland and, consequently, intoxicating all or most of the colony companions^26^. The similarity between the self-grooming behavior with the concentrations of 0.1% and 1% of quinone inside inhibitor fungicide and in the control. Also, this demonstrates that this fungicide not modified the ant workers’ self-cleaning behavior, increasing the insecticide dispersion and contamination”.

The touch behavior as the number of ant workers per group increased, but without differences between the concentrations of quinone inside inhibitor fungicide at 0.1%, 1% and the control, demonstrates that the fungicide did not modify this behavior of the ant workers *A. rubropilosa sexdens*. The non-detection of insecticides or fungicides is important to circulate the active ingredient, contaminating and killing as many workers as possible and, consequently, causing the collapse of the leaf-cutting ant colony^3^.

The death of 100% of *A. sexdens rubropilosa* workers in 21 days with the two concentrations of the quinone inside inhibitor fungicide is unprecedented for a fungicide, probably toxic by contact and ingestion, acting like the insecticide fipronil^15^ with two modes of action increasing the contamination and death of workers after its dispersion by social interactions.

The interactions between *A. sexdens rubropilosa* workers dispersed the fungicide quinone inside inhibitor among them. The mode and mechanism of action of this fungicide on leaf-cutting ant workers is still unknown.

## Data Availability

The datasets used and/or analysed during the current study available from the corresponding author on reasonable request.
